# RTLola on Board: Testing Real Driving Emissions on your Phone

**DOI:** 10.1007/978-3-030-72013-1_20

**Published:** 2021-02-26

**Authors:** Sebastian Biewer, Bernd Finkbeiner, Holger Hermanns, Maximilian A. Köhl, Yannik Schnitzer, Maximilian Schwenger

**Affiliations:** 8 Eindhoven University of Technology, Eindhoven, The Netherlands; 9 Aalborg University, Aalborg East, Denmark; 10 Saarland University, Saarland Informatics Campus, Saarbrücken, Germany; 11 CISPA Helmholtz Center for Information Security, Saarbrücken, Germany; 12 Institute of Intelligent Software, Guangzhou, China

## Abstract

This paper is about shipping runtime verification to the masses. It presents the crucial technology enabling everyday car owners to monitor the behaviour of their cars in-the-wild. Concretely, we present an Android app that deploys rtlola runtime monitors for the purpose of diagnosing automotive exhaust emissions. For this, it harvests the availability of cheap bluetooth adapters to the On-Board-Diagnostics (obd) ports, which are ubiquitous in cars nowadays. We detail its use in the context of Real Driving Emissions (rde) tests and report on sample runs that helped identify violations of the regulatory framework currently valid in the European Union.

## 1 Introduction

In the last decade, far more than 600 million cars have entered the streets worldwide [10]. With very few exceptions, each of these cars is equipped with a standardized On-Board-Diagnostics (OBD [16]) interface. Five years ago it surfaced that many of the cars out there do not adhere to the regulatory framework with which they are supposed to comply. For example, a number of undeniable proofs of tampered emission cleaning systems in passenger cars [5,3,14] are known by now. When this scandal first surfaced, the regulations imposed by the authorities were related to isolated tests carried out under lab-like conditions on chassis dynamometers [20,4]. Since then, there has been a growing understanding that emission and fuel or battery consumption measurements should best take place in a realistic context. Hence, the first test framework for testing on public roads, the *Real Driving Emissions* (RDE) test has been developed [19,17] and is being rolled out for car model approval in Europe and other entities of jurisdiction.

The RDE regulation specifies the conditions under which a car trip qualifies as a valid RDE test. These conditions refer to the trajectory driven, duration,

* This work is partly supported by DFG grant 389792660 as part of TRR 248 – CPEC, by the European Research Council (ERC) grants 683300 (OSARES), 695614 (POWVER), and 966770 (LEOpowver), and by the Key-Area Research and Development Program Grant 2018B010107004 of Guangdong Province.

altitudes, speeds, and on the dynamics of the driving profile [17]. By combining the information available at the OBD port and the position of the car, it is possible to cast RDE testing into a runtime monitoring [21,13,12] problem. Indeed we have shown in earlier work [9] how to formalize the RDE regulations in RTLola [7,8], a real-time extension of the stream-based specification language Lola [6]. Lola combines the ease-of-use of rule-based specification languages with the expressive power of heavy-weight scripting languages or temporal logics. The eponymous framework generates runtime monitors for such specifications, which were successfully deployed, for instance, on unmanned aircraft [18,2].

An official RDE test requires a calibrated *portable emissions measurement system* (PEMS) to be connected to the car's exhaust pipe while driving the test, so as to correctly quantify the amount of exhaust emissions induced. The purchasing costs of a PEMS are in the order of €250,000 which is close to unaffordable even in a research context. However, many car models expose a variety of diagnosis data through OBD and an OBD-to-Bluetooth adapter can be purchased for around €10. The data exposed depends on the type of engine, emission cleaning system, and other components in use. There are several minimal combinations of OBD data giving good approximations of emitted gases. In particular, various car models expose the sensor readings of their after-treatment $NO_x$ sensor deployed at the rear of the exhaust pipe.

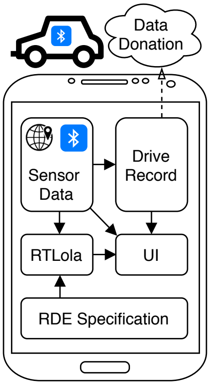

Fig. 1: LolaDrives

*Contribution.* This paper presents LolaDrives, an Android app enabling car owners to carry out real driving emission tests with little investment. Prerequisites are (i) an Android phone, (ii) an OBD-to-Bluetooth adapter, and (iii) a car model that does indeed expose the needed values via OBD. If the latter is not the case, the app can still serve the user as a convenient personal monitoring and logging device for the many quantities exposed while driving.

A structural overview of LolaDrives is depicted in Figure 1. At the core of the app is an Android version of the RTLola engine [7]. The engine is strictly separated from the data acquisition and the RTLola RDE specification. This separation will make it possible to reuse the approach in other runtime monitoring contexts, be it of espresso machines via USB, or drones via Wi-Fi. In both cases, it would especially be the specification in RTLola that needs to change, not the engine. Car sensor data is acquired via Bluetooth from the OBD device, and combined with location data provided by Android's GPS service. The data streams are recorded for later diagnosis. Anticipating future application scenarios involving crowd sourcing car data, we advertise the app as part of a *car data platform* (CDP), which includes an upload facility for donating drive records. While driving, the app's user interface (UI) displays diagnostic information to the user, both regarding the correct execution of an RDE test drive and the car's emission data. We will detail the separate components of the app next.

Notably, the lack of any calibration and the unknown precision of the data exposed by the car manufacturer via OBD make it impossible to consider the RDE test results reported by LolaDrives as anything more than indicators of the car's RDE behaviour in a legal sense.

## 2 RDE Monitoring on Android

The primary feature of LolaDrives is to monitor the progress of an RDE test drive. For this, it uses the RTLOLA monitoring framework. This bridges the gap between formally sound concepts and every-day use cases. While RTLOLA does target a broad audience, that audience is still intended to be expert users rather than the general public. It requires users to execute three tasks: provide a formal specification of the intended behaviour, supply input data, and interpret the monitor's output. LolaDrives reduces these tasks to minimal action points for end-users.

*Specification.* No end-user input is required with respect to the RTLOLA specification. The definition of what is a valid RDE test is fixed [9] and strictly follows the constraints imposed by the regulation issued by the European Commission [17]. These constraints concern the driving behaviour and layout of the route. Some of them apply universally, e.g., the ambient temperature must range between 273 K and 303 K. For others, the RDE regulation differentiates three environments: urban, rural, and motorway with different environments imposing different restrictions on the car, such as an average velocity between 15 and 40 km/h in an urban environment. A *segment* refers to all parts of the test drive in which the car operates in a certain environment. While segments may be interrupted, each one needs to occupy a specific share of the total distance travelled.

*Input Data Provision.* LolaDrives uses sensor readings provided over the OBD interface as input data. The user only has to plug the OBD-to-Bluetooth adapter in the respective port at (or close to) the dashboard of her car and pair it with her phone. The car then automatically transmits data to the phone while driving.

*Interpretation of Output.* While driving, LolaDrives assists the user in the critical task of satisfying all the constraints that make up a valid RDE. It provides feedback on the driving behaviour indicating which requirements on the test are satisfied to what extent, and which still need attention. Furthermore, it evaluates the measured exhaust data and informs the user of whether or not the car violates emission regulations. Both of these tasks require an online analysis of driving data. For this analysis, LolaDrives uses the RTLOLA monitoring framework.

*Foundational Underpinning.* RTLOLA [8,7] is a stream-based runtime verification framework. The RTLOLA monitor analyses sequences of input data to assess whether or not the system complies with the specification. The specification language has a formal semantics which enables devising provably correct monitoring algorithms [15].

An RTLOLA specification consists of input stream declarations where each input stream corresponds to a source of input data such as the $NO_x$ sensor of the car. Output stream declarations then spell out how to filter and refine the input data. For this, RTLOLA provides primitives for complex analyses such as sliding window aggregation for common aggregation functions. Further, the specification contains binary trigger conditions. The satisfaction of such a condition constitutes a violation of the specification and prompts the monitor to immediately relay a warning to the user. The following snippet is an extract of an RTLOLA specification for RDE test drives [11]:

```
input velo_kmph, accel_mpss: Float64
output is_rural := ... output rural_avg_velo := ...
output rural_dyn : Float64 @1Hz filter: is_rural := velo_kmph *
    accel_mpss / 3.6
output rural_pctl_dyn : Float64 @1Hz :=
   rural_dyn.aggregate(over: 7200, using: pctl(95)).defaults(to: 0.0)
trigger rural_pctl_dyn > (0.136 * rural_avg_velo + 14.44)
   ∧ rural_avg_velo <= 74.6
```

This specification fragment checks whether the car complies with the RDE regulations regarding the driving dynamics in the rural segment[4]. The first line declares two input streams representing the velocity in $\mathrm{km\,h^{-1}}$ and acceleration in $\mathrm{m\,s^{-2}}$ supplied by the car. The third line computes the dynamics in $\mathrm{m^2\,s^{-3}}$, by multiplying the velocity and acceleration. The regulations then demand that the $95^{\text{th}}$ percentile of the dynamics are no greater than $0.136 \cdot v_{avg} + 14.44$ where $v_{avg}$ is the average velocity of the vehicle. The computation of the velocity and the dynamics only consider sensor readings obtained while in the rural segment. The full specifications are publicly available [1]. Note that while the specification is relatively easy to design and understand for computer scientists and engineers, it exceeds the expertise expectable of laymen users. However, it is not necessary for them to be confronted with the full potential of the language because LOLADRIVES comes preconfigured with a set of RDE-specific specifications.

As can be seen, the requirements on the end-user are minimal. Thus, the setup enables users to conduct RDE test drives and assess the emission-behaviour of their cars without requiring them to understand the underlying technology.

## 3 User Experience

This section discusses the user perspective on LOLADRIVES. After a general overview, we report on the use of LOLADRIVES for conducting RDE test drives with a rented vehicle (the precise car model being unknown upfront).

*Overview.* The preparation of the test requires the user to plug the OBD-adapter into the OBD-port of the car. After starting car and app, LOLADRIVES receives data packets and determines the sensor profile of the car, assuming phone and adapter are paired via Bluetooth. Some sensor profiles provide insufficient data to conduct an RDE test drive. In this case, the app is still convenient to use for real-time displaying and logging the available data regardless of RDE regulations,

[4] See Annex IIIA, Appendix 7a, 3.1.3 in the EU regulations [17].

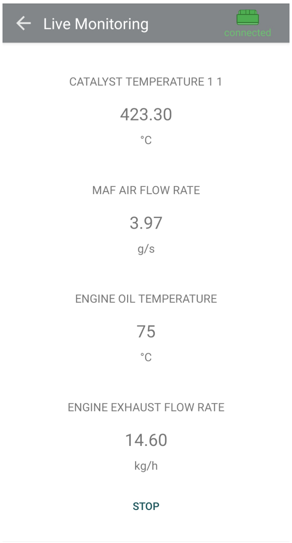

(a) Diagnostics view displays the most recent diagnostics information.

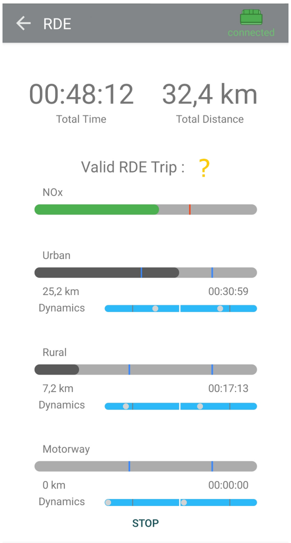

(b) RDE progress view displays current state parameters of the test drive.

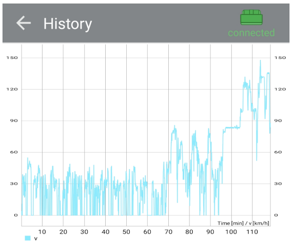

(c) Replay view displays data plotted against time.

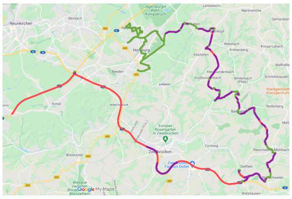

(d) Map of the second RDE test drive.

Fig. 2: UI of LolaDrives displaying different views and a map of a test route.

see Figure 2a. If the data suffices, the app selects an appropriate specification and initializes the RTLola monitor. LolaDrives then starts filtering and visualising the data output and trigger notifications provided by the monitor.

After successful setup, the UI switches to an RDE guiding view (Figure 2b). From top to bottom, it shows the total time, which must be between 90 and 120 min to finish the test, and the total distance travelled. The next line indicates the current state of the conditions for a valid RDE test drive disregarding emission data. In the screenshot, the drive is still in progress and inconclusive, indicated by the question mark. Instead, the UI can also indicate success or failure. The latter verdict can occur far before the time limit is reached, caused by an irrecoverable situation such as transgression of the 160 $\mathrm{km\,h^{-1}}$ speed limit. Note that the indicator reports the *current* status if the test drive were to end in this moment. Together with the regulatory constraints, this implies that the current verdict can alternate between success and failure from minute 90 to 120. As there is no specific point in time when the test ends, the app continues to compute statistics until the tester manually stops it or the 120 min mark is reached. Beneath the status indicator is the green $NO_x$ bar displaying the total $NO_x$ emissions. The vertical red bar denotes the permitted threshold of 168 $\mathrm{mg\,km^{-1}}$.

The next three UI groups represent the progress in each of the distinct segments: urban, rural, and motorway. Each group consists of two horizontal bars. The gray progress bar displays the distance covered in the respective segment. The vertical blue indicators denote lower and upper bounds as per official regulation, for an expected trip length of 83 km. The blue bar below the gray one

| | Drive 1 | | | Drive 2 | | |
|---|---|---|---|---|---|---|
| | Distance [km] | $NO_x$ [mg/km] | $CO_2$ [g/km] | Distance [km] | $NO_x$ [mg/km] | $CO_2$ [g/km] |
| Urban | 35.45 | 137 | 222 | 37.46 | 102 | 251 |
| Rural | 22.33 | 305 | 154 | 27.40 | 90 | 172 |
| Motorway | 26.10 | 241 | 153 | 25.37 | 105 | 175 |
| Total | 83.88 | 214 | 183 | 90.22 | 99 | 205 |

Table 1: Aggregation of the emission data based on the CDP.

illustrates two different metrics for the driving dynamics. Both dots need to remain below/above their thresholds. A more aggressive acceleration behaviour shifts the dots to the right and a passive driving style to the left.

*Test Drive.* The technical framework and visual feedback of the app were tested in two RDE test drives. Both tests were conducted with an Audi A6 Avant 45-TDI hybrid diesel, which is approved as Euro 6d-TEMP (DG) with an $NO_x$ threshold of 80 mg km$^{-1}$ under lab conditions and 168 mg km$^{-1}$ for RDE conditions. Among the diagnosis parameters available within this car are vehicle and engine speed, ambient temperature, engine fuel rate, mass air flow, and two $NO_x$-sensors—one in front and one behind the emission cleaning system in the exhaust pipe. With this data, exhaust mass flow and fuel consumption can be computed, from which the total amounts of $NO_x$ and $CO_2$ can be derived [11]. In both drives, the driving dynamics were close to the allowed maximum, in the first test below and in the second test above the threshold, so the second test drive did not result in a valid RDE test. In both cases, the app correctly confirmed the satisfaction and violation of the RDE criteria. In the first drive, the app reported an average $NO_x$ emission of 214 mg km$^{-1}$. This constitutes a violation of the regulation.

The app also allows for inspection of the driving data in a plotted form (Figure 2c). Figure 2d shows the route of an RDE test drive. The first half of the time constituted the urban segment (green). The next 30-40% of the test mainly consisted of the rural segment (purple) followed by the motorway segment (red).

*Data Harvesting.* For further analysis, data can be uploaded to a cloud storage which is part of the car data platform (CDP). This platform provides a uniform way to harvest data by specifying a format for collection, analysis, and exchange of this data. CDP builds upon a JSON format (`https://json-schema.org/`) containing timestamped events such as an OBD response, including its raw payload. As an example, the data presented in Table 1 is an aggregation of the RDE test drives mentioned above obtained by post-processing the data.

## 4 Conclusion

LolaDrives pushes runtime verification technology into cars and phones of everyday users. The app is available in *Google Play* [1]; a version for iOS is already initiated. Moreover, the car data platform constitutes a crowd-sourcing initiative for car data with the intention to enable large scale analyses of emission data beyond a single trip and a single car model.
